# Sample size calculation for prevalence studies using Scalex and ScalaR calculators

**DOI:** 10.1186/s12874-022-01694-7

**Published:** 2022-07-30

**Authors:** Lin Naing, Rusli Bin Nordin, Hanif Abdul Rahman, Yuwadi Thein Naing

**Affiliations:** 1 PAPRSB Institute of Health Sciences, Universiti Brunei Darussalam, Jalan Tungku Link, Brunei-Muara BE3119, Gadong, Brunei Darussalam; 2 Faculty of Medicine, Bioscience and Nursing, MAHSA University, Bandar Saujana Putra, Jenjarom, Selangor Malaysia; 3 Centre of Advanced Research (CARe), Universiti Brunei Darussalam, Gadong, Brunei Darussalam; 4 School of Nursing and Statistics Online Computational Resource (SOCR), University of Michigan, Ann Arbor, MI USA; 5 Graduate Student, Asia Pacific University of Technology and Innovation, Kuala Lumpur, Malaysia

**Keywords:** Sample size, Calculator, Single proportion, Prevalence studies

## Abstract

**Background:**

Although books and articles guiding the methods of sample size calculation for prevalence studies are available, we aim to guide, assist and report sample size calculation using the present calculators.

**Results:**

We present and discuss four parameters (namely level of confidence, precision, variability of the data, and anticipated loss) required for sample size calculation for prevalence studies. Choosing correct parameters with proper understanding, and reporting issues are mainly discussed. We demonstrate the use of a purposely-designed calculators that assist users to make proper informed-decision and prepare appropriate report.

**Conclusion:**

Two calculators can be used with free software (Spreadsheet and RStudio) that benefit researchers with limited resources. It will, hopefully, minimize the errors in parameter selection, calculation, and reporting. The calculators are available at: (https://sites.google.com/view/sr-ln/ssc).

## Background

In quantitative research, when we take a sample from a study population or eligible population in order to save our resources, there are two important statistical processes namely using a probability sampling method (commonly known as “random sampling”) [1], and calculating an appropriate sample size [2]. Both are equally important to ensure a good representative sample for the study population.

As we need a specific statistical analysis for a specific research objective, we also need a specific sample size calculation method for a specific research objective. Even if two research objectives may require a similar statistical analysis, the sample size might be different depending on the parameters that we use for the calculation. In this paper, we focus on the objective that estimates a prevalence or proportion, for example, to estimate the prevalence of obesity, the prevalence of smoking, the prevalence of heart disease, diabetes mellitus or any other diseases of a study population. The method in this paper will not be suitable for other type of objectives such as estimating mean, comparing means, comparing proportions or regression analyses.

Books [3, 4] and published articles [5, 6] guiding the methods of sample size calculation for prevalence studies are available. Nevertheless, we observed that several parts of the sample size calculation process can be guided by a software or calculator and it can prevent incorrect calculation, incorrect use of formula, incorrect parameters, and incomplete sample size reporting.

Sample size softwares and calculators are extremely helpful that are available through commercial licenses such as Power Analysis & Sample Size (PASS) [7], or via freely available softwares such as Epitools [8] and the “presize” package in R [9]. However, there are a lot of confusion that still exists, that resulted in users incorrectly calculating sample size of their studies [10, 11] especially the erroneous notion that one blanket formula can be used for all study designs [6]. In addition, users are expected to have some statistical knowledge to calculate and report the sample size calculation. Incorrect sample size calculation could introduce statistical errors that give rise to inaccurate results, which could be serious, particularly in medical research where evidences from these research studies are cornerstones of medical practices [12, 13]. Many reasons could be attributed to these confusion, inaccuracy, and misunderstanding, in particular, the complexity of available softwares and corresponding guidelines [13].

Therefore, in this paper, we are addressing these issues by introducing a user-friendly Excel calculator that guides users to use the correct method and parameters step-by-step. This calculator also generates a publication-style report of adequate sample size for users’ study. We believe that, this will improve sample size calculation in future prevalence studies in medical and health sciences.

## Implementation

### Method to calculate sample size

For an objective that estimates a prevalence, the sample size calculation formula is fairly simple and available in a number of books.

The following formula [2] shall be used:

$$n=\frac{{Z}^{2}P\left(1-P\right)}{{d}^{2}}$$

where *n* = Sample size,

*Z* = Z statistic for a level of confidence (1.96 for 95% confidence level),

*P* = Expected prevalence or proportion, and.

*d* = Precision.

However, we do not encourage researchers to use formula as it could have human error in manual calculation. We can use available softwares, and concentrate on carefully choosing appropriate parameters for the calculation.

### Appropriately choosing parameters

The above formula indicates three parameters to be determined.

#### Parameter 1: level of confidence

When we take a sample but wish to know about the population (such as prevalence of smoking) from where the sample is taken, we will not know the exact prevalence of the population as we do not study all members of the population. However, the sample study gives us an estimation which has lower and upper limits (informally ‘a range’, but we call ‘interval’ in Statistics) for the population prevalence. We normally calculate these lower and upper limits or an interval with a certain level of confidence. Commonly used or almost always used “level of confidence” for these intervals or estimates, is 95% (which we called 95% confidence interval, CI) in medical and health fields. In addition, most data analysis softwares give the results with 95% CIs by default. For these reasons, and also to minimize users’ error by non-statisticians, we have fixed the level of confidence as 95% without giving users’ choice in these presented calculators.

#### Parameter 2: precision

As mentioned above, we will not know the exact prevalence of the population as we do not study all members of the population. Therefore, the prevalence we calculate from the sample could deviate (error) from the population prevalence. We call this deviation as sampling error. We also know that, the larger the sample size, the smaller the errors in estimation. The errors are calculated as precision or also known as ‘margin of error’.

Practically, the precision reflects the width of 95% confidence interval. If we decide to choose an absolute precision of ± 2% in estimating a prevalence, we should expect, in the result, the width of 95% CI as 4% (example: 95% CI: 23%, 27%). If the absolute precision is ± 5% in estimating a prevalence, we should expect, in the result, the width of 95% CI as 10% (example: 95% CI: 20%, 30%). The width of the CI is twice that of the precision. Details are presented in Table 1.

**Table 1:** Relationship between Precision and width of Confidence Interval (CI)

Prevalence in Sample	Absolute Precision	95% CI for Population	CI Width	Required Sample size
25%	± 2%	(23%, 27%)	4%	1801
25%	± 5%	(20%, 30%)	10%	289
25%	± 10%	(15%, 35%)	20%	73
30%	± 2%	(28%, 32%)	4%	2017
30%	± 5%	(25%, 35%)	10%	323
30%	± 10%	(20%, 40%)	20%	81

It is an opportunity for researchers to decide the precision (margin of error) and the width of the CI that they wish to see in the results. Normally, researchers wish to have narrower width of CI but the narrower it is, the more expensive (bigger sample size) it is going to be. Even if researchers decide to go for a smaller sample size, the researchers can also foresee or appreciate how poor CI width is going to be in their results. Therefore, this is an informed decision to be made by researchers.

Practically, we give some recommendations for choosing a precision value (Table 2). In general, well-funded studies or large scale studies, aiming to gain attention from policy makers, should aim for a precision of 2 to 3%, whereas small scale (or poorly-funded studies), for example, undergraduate or master student research projects, may consider a precision of 4 to 5%. If the precision is larger than 5% (such as 10%), due to limited resources, researchers should consider the study as a preliminary study.

**Table 2:** Recommended precision for expected prevalence

	EP	Recommended Precision			
		Large Scale	Small Scale	Preliminary Study	Remark
1	10 to 90%	2 ~ 3%	4 ~ 5%	> 5%	
2	< 10%	0.25*EP	0.50*EP	> 0.50*EP	Cannot be equal to EP or larger
	*e.g*. 4%	1%	2%	> 2%	Cannot be 4% or larger
3	> 90%	0.25*(100-EP)	0.5*(100-EP)	> 0.5*(100-EP)	Cannot be (100-EP) or larger
	*e.g*. 95%	1.25%	2.5%	> 2.5%	Cannot be 5% or larger

*EP* Expected Prevalence in percent

However, the above recommendation applies to the expected prevalence of 10 to 90%. When the expected prevalence is too small (less than 10%) or too large (more than 90%), we need to apply much smaller precision. It is obvious that a precision of 5% is possible for an expected prevalence of 50%, but 5% precision is totally inappropriate for an expected prevalence of 2%.

We present details of precision for expected prevalence with examples in Table 2.

#### Parameter 3: variability of the data

The larger the variation the data has, the larger is the sample size needed. This relationship can be explained in a simple analogy. When we cook soup and near to the finish, we stir it well before we taste. We always need a very small amount (small sample size) to taste because we stir it well and the variation is almost zero.

**Fig. 1:**
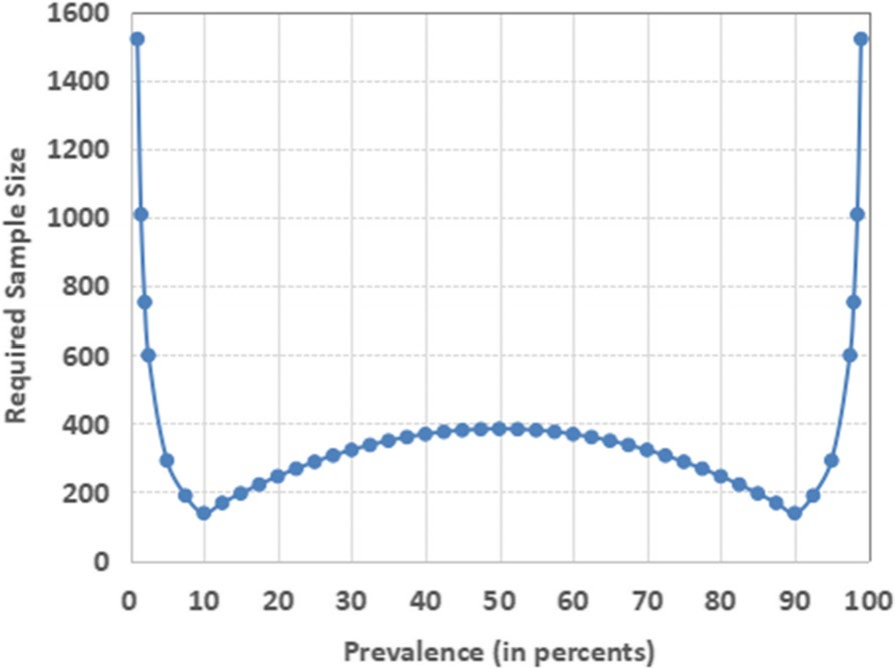
Prevalence and Effect on Sample Size

Practically, in estimating prevalence, the prevalence has effect on this variation and therefore effect on the required sample size. The relationship of prevalence and the sample size is presented in Fig. 1.

Obviously, it is the research objective to estimate the prevalence and researchers do not know this prevalence. Therefore, to calculate sample size, we normally find it out from most recent published studies with similar study population. If we cannot find suitable studies in the literature, we may consider to conduct a pilot study.

When we find multiple suitable prevalence from the literature, for example ranging from 15 to 30%, we should use the prevalence giving the highest sample size (in this case, 30%) in accordance with Fig. 1 that shows 30% will require the largest sample size in that range of 15 to 30% prevalence. Similarly, if the prevalence ranges from 60 to 80% in the recent literature, we should use 60% as it requires the largest sample size in that range.

We would like to caution that some books or guidelines suggest to use expected prevalence 50% if we could not get the prevalence at all [2, 14, 15]. We discourage this practice. In Fig. 1, we should note that the prevalence of 50% will produce the largest sample size only within the range of 10 and 90% of the prevalence. The required sample size is much higher in the region below 10 and above 90%. Therefore, a short cut of prevalence 50% should not be used. It is best to calculate the sample size with appropriate expected prevalence. Researchers may find possible range of expected prevalence and apply the recommendation in the previous paragraph.

For this illustration, we have drawn Fig. 1 using precision for small scale study (Table 2). It means that we use the precision of fixed 5% for the expected prevalence between 10 and 90%, half of the expected prevalence for the expected prevalence less than 10%, and half of the (100 minus expected prevalence) for the expected prevalence larger than 90%.

#### Parameter 4: anticipated loss

We always have loss in sample size during the research process due to several reasons, such as non-response, incomplete data, loss-to-follow up, etc. Researchers should estimate the loss with their past experience, and inflate the sample size in calculation accordingly. These losses (especially, non-response, incomplete data, and loss-to-follow up) are very much related to research areas (for example, non-response rate could be higher if we study sexual issues or other sensitive issues) and population that researchers intend to study. Therefore, we recommend researchers to use non-response rates of previous studies of similar research areas and in similar populations.

Although we can put any per cent of the potential loss and inflate the sample size, it doesn’t guarantee that the calculated sample size is valid in terms of representative sample. In general, we would recommend that less than 10% loss would be an acceptable loss. However, there are different opinions on the acceptable per cent of loss or attrition [16] depending on the type of studies. At least, it is important to note that the higher the loss or attrition, the larger will be the compromise on the validity of the results.

### Sample size calculation report

The report of sample size should be reproducible. It means that all parameters used must be reported. There are four parameters namely, level of confidence (mostly 95%), expected prevalence (mostly from literature or pilot study), the precision or margin of error of estimate (decision by researchers) and anticipated loss (experience of researchers) used in the calculation. We should also include the name of the software or calculator with proper reference. Scalex SP calculator has incorporated the draft report for the user to copy and use. It ensures all necessary parameters used are included in the report.

## Results and discussion

### Demonstration of Scalex SP and ScalaR calculator

#### Simple three steps for Scalex SP

Basically, the Scalex SP calculator (Scalex stands for ‘Sample Size Calculator using Excel’, and SP stands for ‘Single Proportion’) (available at: https://sites.google.com/view/sr-ln/ssc) guides the users in three steps:

Step 1: to type in “Expected Prevalence” in terms of per cent (> 0 to < 100).

Step 2: to type in “Anticipated Loss” in terms of per cent (0 to < 100).

Step 3: to decide and type in the precision of user choice after going through the Sample Size Table. Users may type a precision which is not listed in the table (such as ± 2.5%). Then, Scalex SP will give a draft report for the user.

Major advantage of the Scalex SP calculator is that, it gives users Sample Size Table (Fig. 3) in which users can appreciate sample sizes for a range of precision, and appreciate or foresee the CIs in their results. Therefore, it helps users in decision making of selecting precision considering available resources.

#### Example using Scalex SP

We are going to conduct a study to estimate the prevalence of obesity among secondary school children in a district. We managed to find the expected prevalence in the literature as 30%.

**Fig. 2:**
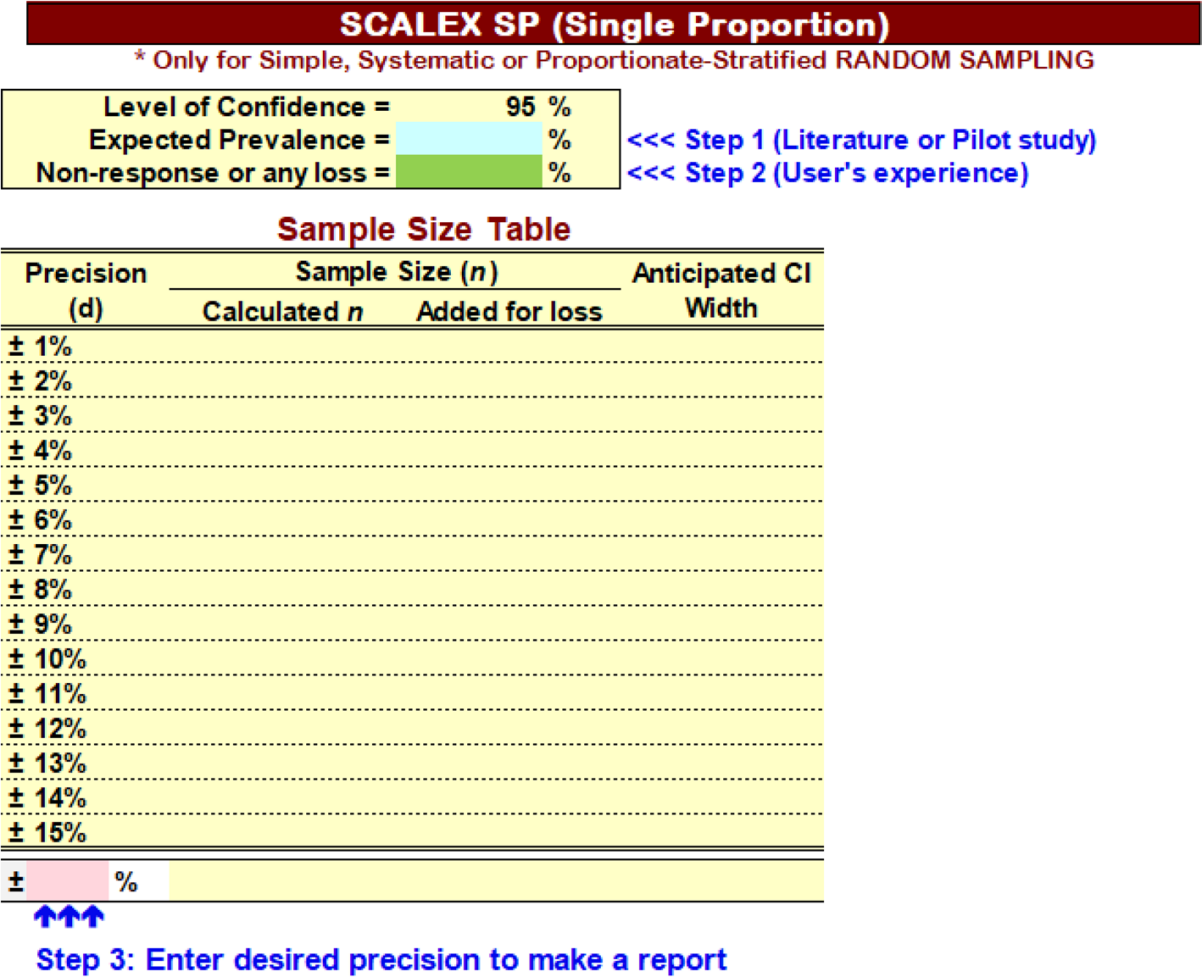
Scalex SP interface for Step 1, 2 and 3

**Fig. 3:**
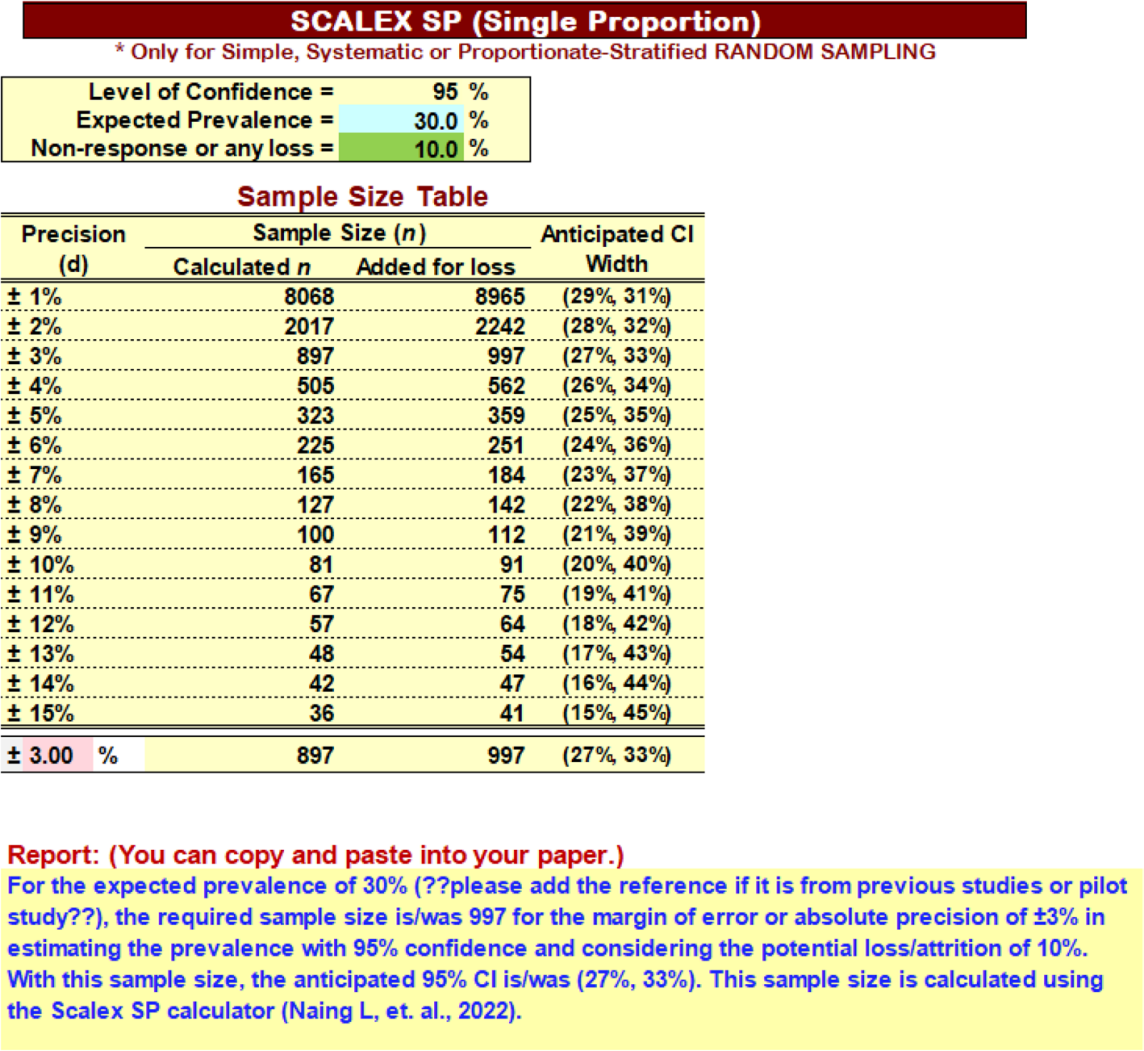
Scalex SP with Report

When we start the Scalex SP, we see the interface as in Fig. 2. Then, we fill 30 (30%) for Expected Prevalence. As we experienced 10% non-response in this study population in previous studies, we fill 10% loss (see Fig. 3).

Then, sample sizes given for various precisions are reviewed and we decide to use ± 3% precision as it gives us an acceptable width of 95% CI (27%, 33%), and the sample size (*n* = 997) is possible to manage.

Then, we fill in 3 (3%) in Step 3, and Scalex SP gives the draft report as in Fig. 3.

#### ScalaR SP programme for R users

**Fig. 4:**
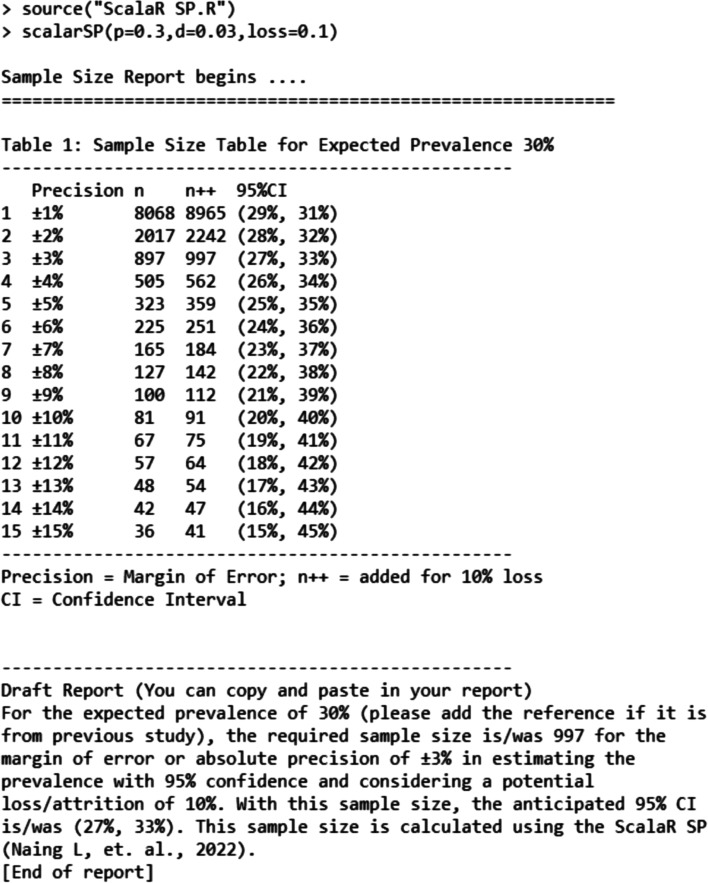
ScalaR SP—with report

Authors have written R Script (ScalaR SP.R) and with two command lines as in Fig. 4 (this Script file must be stored at “Working Directory”), will give the same output as Scalex SP.

(available at: https://sites.google.com/view/sr-ln/ssc).

Example of R command as follows:

 > ScalarSP(*p* = 0.3, d = 0.03, loss = 0.1).

*p* = expected prevalence.

d = precision or margin of error.

loss = anticipated loss or attrition of sample size.

#### Other issues

The Scalex calculator is for studies using the specific sampling method such as simple random sampling, systematic sampling, and proportionate-stratified random sampling. For other sampling methods, the calculated sample size should be multiplied with the design effect [14]. Estimating design effect could be from the literature if it is reported in the previous similar studies. If not, it is a complicated procedure involving data simulation.

#### Limitation of the presented calculators

The formula used in these calculators (reported in Para 2 above) assumes that the population is unknown and large. If the population is known, the required sample size could be smaller by using a different formula which has population size in the formula. However, if we use the formula with population size and obtain smaller sample size, researchers should analyse the data using ‘finite population correction’ and ‘survey data analysis method’ [17] instead of standard statistical analyses, to obtain valid results. Therefore, we consider a safer approach, that is, assuming that the population size is unknown both in calculating sample size and also later in data analyses. Therefore, it could be a limitation, if one would like to calculate a sample size with known population size and also using ‘finite population correction’ in their data analyses.

The presented calculators have been designed using Wald’s confidence interval. The limitation of this confidence interval is that, it could go below 0% or above 100% in the confidence intervals if the users specify precision inappropriately in relation to the expected prevalence. Though we could give users a choice to consider other methods of confidence interval such as exact confidence interval, logit-confidence interval, etc. we prevent this issue by recommending the use of appropriate precision in Implementation Paragraph 2.1.2 and Table 2. We consider this would be a more intuitive approach especially for users with limited statistical knowledge or skills. In any case, with a single method of confidence interval (Wald), we wish to report this limitation for the presented calculators.

## Conclusions

With technological advancement, researchers should not calculate sample sizes manually. The software or calculators should help researchers minimize possible error in calculation and also to assist in reporting. However, the use of correct parameters still remains as the responsibility of users. In addition, calculators using free software, will benefit researchers who have limited resources.

The presented calculators, designed for prevalence studies, is available at: (https://sites.google.com/view/sr-ln/ssc) for public without asking permission. Authors will continue to use Scalex calculator for other type of studies in the near future.

The presented calculators are beneficial as the calculators incorporate non-response or other loss, indicate the anticipated 95% CI, give a list of sample sizes for a range of precisions therefore, guide to make informed decision for precision, and finally draft a sample size calculation report for scientific reporting.

This paper also includes a number of cautions and recommendations for selecting parameters, especially expected prevalence, precision, and anticipated loss, so that researchers can conduct prevalence studies with more appropriate sample sizes.

### Availability and requirements

Scalex SP calculator.

Project name: sample size calculator project.

Project home page: https://sites.google.com/view/sr-ln/ssc

Operating system(s): Windows.

Programming language: Excel-based.

License: no license required.

Any restrictions to use by non-academics: No restriction.

ScalaR calculator.

Project name: sample size calculator project.

Project home page: https://sites.google.com/view/sr-ln/ssc

Operating system(s): Windows.

Programming language: R language.

License: no license required.

Any restrictions to use by non-academics: No restriction.

## Data Availability

This paper doesn’t involve data. However, the free calculator is available here: (https://sites.google.com/view/sr-ln/ssc).

## Abbreviations

Scalex SP: Sample Size Calculator using Excel for Single Proportion
ScalaR SP: Sample Size Calculator using R & RStudio for Single Proportion
PASS: Power Analysis and Sample Size
CI: Confidence Interval
*n*: Sample Size
*Z*: *Z* Statistic
*P*: Expected prevalence or proportion
*d*: Precision
